# Robust normalization protocols for multiplexed fluorescence bioimage analysis

**DOI:** 10.1186/s13040-016-0088-2

**Published:** 2016-03-05

**Authors:** Shan E Ahmed Raza, Daniel Langenkämper, Korsuk Sirinukunwattana, David Epstein, Tim W. Nattkemper, Nasir M. Rajpoot

**Affiliations:** Department of Computer Science, University of Warwick, Coventry, CV4 7AL UK; Biodata Mining Group, Bielefeld University, Bielefeld, Germany; Mathematics Institute, University of Warwick, Coventry, CV4 7AL UK; Department of Computer Science and Engineering, Qatar University, Doha, Qatar

**Keywords:** Multiplexed fluorescence imaging, Protein signatures, Toponome imaging system, Normalization protocols, Bioimage informatics

## Abstract

study of mapping and interaction of co-localized proteins at a sub-cellular level is important for understanding complex biological phenomena. One of the recent techniques to map co-localized proteins is to use the standard immuno-fluorescence microscopy in a cyclic manner (Nat Biotechnol 24:1270–8, 2006; Proc Natl Acad Sci 110:11982–7, 2013). Unfortunately, these techniques suffer from variability in intensity and positioning of signals from protein markers within a run and across different runs. Therefore, it is necessary to standardize protocols for preprocessing of the multiplexed bioimaging (MBI) data from multiple runs to a comparable scale before any further analysis can be performed on the data. In this paper, we compare various normalization protocols and propose on the basis of the obtained results, a robust normalization technique that produces consistent results on the MBI data collected from different runs using the Toponome Imaging System (TIS). Normalization results produced by the proposed method on a sample TIS data set for colorectal cancer patients were ranked favorably by two pathologists and two biologists. We show that the proposed method produces higher between class Kullback-Leibler (KL) divergence and lower within class KL divergence on a distribution of cell phenotypes from colorectal cancer and histologically normal samples.

**Electronic supplementary material:**

The online version of this article (doi:10.1186/s13040-016-0088-2) contains supplementary material, which is available to authorized users.

## Introduction

The study of co-localized proteins at the subcellular level is key to our understanding of the functional relationships between proteins in abnormal cells in complex diseases such as cancer, as proteins interact together at sub-cellular level to perform cell functions. Different technologies have been developed in the recent years allowing simultaneous imaging of the same tissue specimen with several stains or markers. This makes it possible to study co-localized protein patterns at the cellular and sub-cellular levels, potentially leading to the discovery of functional protein complexes, protein hubs, stem cell niche, interactions between neighboring cells in cancerous tissue, novel cancer subtypes, and multiplex biomarkers for a particular subtype of cancer [1]. Some of the popular multiplexed bioimaging (MBI) techniques are based on immuno-fluorescence microscopy [2–4], mass cytometry [5–7], Raman spectroscopy [8] and Ion-beam imaging [9]. All of these techniques require a standard laboratory procedure to prepare a sample before data acquisition. To avoid the garbage-in garbage-out (GIGO) phenomenon in analysis, preprocessing of the MBI data is as important as standardization of laboratory procedures [10]. The aim of the overall pre-processing of the MBI data (i.e. a set of n MBIs obtained for n visual fields per sample) is to align and normalize the data so the signals of one MBI for protein marker A can be compared to the signals in another MBI for protein B. Since the co-location of the signals and signal intensities are of primary concern, the MBIs must be aligned in two domains, the spatial domain (a problem that is usually referred to as the signal registration) and the intensity domain (the problem of signal normalization), so that the signal intensities of any given sub-cellular location or ROIs (regions of interest) can be compared between different data sets.

In this paper, we focus on the standardization of normalization protocols for the MBI data collected from fluorescence microscopy based systems with particular emphasis on data generated from the Toponome Imaging System (TIS). This technology has also been referred to as MELC (multi-epitope-ligand cartography [2] or ICM (imaging cycler microscope) [2, 11]. Like other multiplexing technologies, TIS has the capability to simultaneously image multiple protein markers at subcellular level by staining the tissue with fluorescent tags and bleaching in a cyclic manner [2, 11, 12]. The strength of TIS lies in its ability to map co-localized tags on the tissue specimen *in situ* without harming / destroying the tissue. This multiplexing technology has been used to study functional protein networks in different cancers [11] and to co-map dozens of different receptor protein clusters on the surface of peripheral human blood lymphocytes [13]. Recently, sophisticated analytical tools and advanced algorithms have been developed to spatially align TIS images [14], perform cell segmentation [15–18], phenotype cells based on their protein expression profiles [19], visually explore the spatial features of protein co-location [20, 21] and analyze protein networks localized to individual cells without relying on raw pixel intensities [22] as opposed to mapping of protein clusters on pixels as in [2]. The quality of images produced by TIS (and also by multiplexing technologies), varies depending on the quality, quantity and concentration of the tag applied to the tissue and also on exposure time, LED intensity and inherent limitations of the camera capturing the signal. In order to overcome the variation in captured images from various tags across different runs, it is necessary to standardize the methods used for qualitative and quantitative assessment of protein expression profiles of individual cells in the tissue specimen. The goal of this work is to investigate normalization methods that can produce consistent visualization for heterogeneous protein signatures across a range of tissue specimens used in biological experiments. The consistency in visualization is one way of observing the data to produce consistent data for analysis algorithms to produce robust and repeatable results across various runs. We show in our experiments that with the proposed normalization protocols we can increase the separation between the data from different types of tissue and reduce the separation within the same type.

The most commonly used approach to analyze TIS image data is to first convert the image pixels to binary values based on a manually selected threshold after background subtraction [13, 23]. The binary values are then grouped together to form combinatorial molecular patterns (CMPs). The similarity mapping approach (SIM) [11] is similar to binarization, as it allows the user to pick a particular pixel and then analyze all the pixels which show similar profile in the data set. Conventional approaches to analyze the TIS image data rely on raw intensities of pixel values, though analytical techniques employing pairwise dependence between protein markers localized to cells have recently been proposed [22]. Analysis based on intensity values is prone to error and may produce non-reproducible results if there is no standard method to normalize the data to a comparable scale. This has been shown for MBIs obtained using other technologies, such as the matrix-assisted laser desorption (MALDI) technique [24] and mass cytometry [25] and is what one must expect in the case of TIS as well.

In this paper, we compare eight different normalization protocols, along with the raw pixel intensity data (protocol R) and suggest a robust normalization method that is relatively insensitive to intensity variation of fluorescence microscopy images corresponding to various tags among different runs and is responsive to the underlying protein signatures of various tissue constituents. The proposed normalization method provides the best contrast and inter-class stability across different runs when compared to eight different normalization protocols. Here by best, we mean as judged by experts, two pathologists and two biologists, each blinded to the others’ rankings based on the following criteria:

A) Inter-class contrast: Different tissue classes should be represented by different colors.
B) Intra-class homogeneity: Pseudo-color for two regions showing the same tissue class should be identical across the different runs.
C) Inter-sample homogeneity: The pseudo-color contrast features for different tissues should be the same for different samples. If an interesting spatial distribution pattern “pops out” in one visualization, it should do as well in the others too if it is present there as well.

We also test the quality of the data produced using different normalization protocols by quantitatively calculating KL divergence between the data from different type of tissues. The normalization methods presented in this paper are not limited to colorectal TIS MBI data and could be applicable to image data produced by other multiplexed imaging technologies such as the MxIF [3] and tissue samples.

## Materials and methods

The data used in this study consisted of ten different sets of MBIs, four of them from colorectal cancer (CRC) tissue specimens and the remaining six from histologically normal tissue. The images were captured using a TIS machine installed at the University of Warwick following the protocols described in [13]. Before surgery at the George Eliot Hospital, Nuneaton, UK, patients gave written consent for the use of their tissue for research purposes. The approval for this research was granted by the Warwickshire Local Research Ethics Committee, Warwickshire, UK. After collection, the tissue specimen was fixed in para-formaldehyde solution and after overnight cryo protection in sucrose solution, it was embedded in optical coherence tomography (OCT) blocks and stored frozen. Tissue sections were cut from each block and then air-dried after soaking in ice-cold acetone. Before placing the coverslips for TIS imaging, the tissue specimens were soaked in sterile Phosphate Buffered Saline (PBS), then incubated with normal goat serum in PBS, and then washed in PBS. See [26] for more details. A library of 26 tags consisting of a variety of cell-specific markers, tumor and stem cell markers, the nuclear marker (DAPI), and four PBS control tags was applied to the tissue specimen during the run. Each tag was sequentially applied from the library to the tissue section where an image is acquired before and then again after incubation as described in [2]. Each image was captured at 63× with a spatial resolution of 1,056×1,027 pixels and approximately 206 nm/pixel. The images in each stack were aligned using the registration algorithm specifically designed for the alignment of MBIs generated by the TIS microscope [14].

**Fig. 1:**
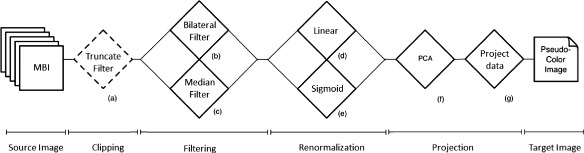
Flowchart of the normalization pipeline. Workflow chart of the normalization pipeline for multiplexed bioimages (MBI). The dashed boxes show non-mandatory processes and the solid boxes show mandatory process. For a detailed view, please see the Materials and methods section

In this paper, we investigate eight different normalization protocols (I-VIII) and the raw intensity MBI (protocol R), as described in the remainder of this section. Figure 1 shows a unified workflow of the normalization pipeline for MBI, for all the eight protocols. The dashed boxes show optional processing modules and the solid boxes show mandatory process in the pipeline. Using different combination of these methods, we established and compared all the protocols as denoted in Table 1.

**Table 1:** Normalization protocols as combinations of clipping, filtering, and renormalization methods

	Clipping (a)	Filtering	Renormalization
R	No	No	No
I	No	bilateral filter (b)	linear renormalization (d)
II	No	bilateral filter (b)	sigmoid renormalization (e)
III	Yes	bilateral filter (b)	linear renormalization (d)
IV	Yes	bilateral filter (b)	sigmoid renormalization (e)
V	No	median filter (c)	linear renormalization (d)
VI	No	median filter (c)	sigmoid renormalization (e)
VII	Yes	median filter (c)	linear renormalization (d)
VIII	Yes	median filter (c)	sigmoid renormalization (e)

For the remainder of this paper, the following notation is used. Let $\mathbb {D}$ denote the domain, for example the set of possible 12-bit intensities that can be measured in a single pixel *p* for a single tag *t*. The width and height in pixels of an image are denoted by *w* and *h* respectively. So the set of all conceivable images for a single tag is ${{\mathbb {D}}^{w\times h}}$. Let ${{I}_{t}}\in {{\mathbb {D}}^{w\times h}}$ denote the intensity image corresponding to the tag *t* in a given MBI stack, and the entire stack consisting of images acquired using *N* tags be denoted by ${{\left ({{I}_{t}}\in {{\mathbb {D}}^{w\times h}}\right)}_{t=1,\ldots,N}}$. We denote by *f*_*t*_(*p*) the intensity of pixel location *p* in image *I*_*t*_.

### The truncate filter (a)

We first describe the non-mandatory step of clipping or truncation in the normalization pipeline, as shown in Fig. 1. Most of the denoising algorithms assume the underlying noise to be a Gaussian distribution. However, during image acquisition various non-Gaussian signals with impulsive characteristics are added to the image at extreme ends of the image histogram, and these may affect any follow-up analysis. To eliminate the outlier values, we truncate the highest and lowest values per intensity image as recently proposed in [25]

(1) $$\begin{array}{*{20}l} {{\hat{f}}_{t}}(p)= \left\{ \begin{array}{ll} f_{t}^{_{0.01}}, & \text{if}\; {{f}_{t}}(p)<f_{t}^{_{0.01}} \\ f_{t}^{_{99.99}}, & \text{if}\; {{f}_{t}}(p)>f_{t}^{_{99.99}} \\ {{f}_{t}}(p), & \text{otherwise} \\ \end{array} \right. \end{array} $$

with $f_{t}^{_{x}}$ being the *x*-th percentile of *I*_*t*_.

### Bilateral filter (b)

For denoising purposes, we explored two popular options: relatively recent bilateral filtering and the more conventional median filtering. Bilateral filter [27] uses a combination of domain and range filters that give relatively large weight to the pixels of a window in close proximity to the center pixel (whose value is to be smoothed) and having a similar intensity, and relatively small weight for pixels that are at a distance and have different intensities. Let ${{\Omega }_{{{M}_{b}}}}\phantom {\dot {i}\!}$ denote the *M*_*b*_×*M*_*b*_ window with the pixel *p* to be smoothed at the center of the window. Mathematically, the bilateral filter can be written as follows,

(2) $$ \begin{aligned} {{\hat{f}}_{t}}(p) &= \frac{1}{o(p)} \sum\limits_{{{p}^{\prime }}\in {{\Omega}_{{M}_{b}}}}{{f}_{t}}(p) g(p,{{p}^{\prime}}) s(p,{{p}^{\prime}}) \\ g(p,{{p}^{\prime }}) &= {{e}^{-\frac{||p-{{p}^{\prime }}||^{2}}{2{\sigma_{d}^{2}}}}}\\ s(p,{{p}^{\prime }}) &= {{e}^{-\frac{||{{f}_{t}}(p)-{{f}_{t}}({{p}^{\prime}})||^{2}}{2{\sigma_{r}^{2}}}}}\\ o(p) &= \sum\limits_{{{p}^{\prime}}\in {{\Omega }_{{M}_{b}}}} {g}(p,{{p}^{\prime}}) s(p,{{p}^{\prime }}) \\ \end{aligned}  $$

We applied bilateral filter with parameters *M*_*b*_=3, *σ*_*d*_=0.5 and *σ*_*r*_=10 for this work.

### Median filter (c)

The median filter [28] is a popular non-linear filter conventionally used in fluorescence microscopy images. It replaces the intensity value of the center pixel with the median of intensity values of a neighborhood window of the size *M*_*m*_×*M*_*m*_. The median filter has excellent noise-reduction capabilities with good edge preservation particularly in the presence of bipolar and unipolar impulse noise [28]. Mathematically, the median filter can be expressed as follows,

(3) $$\begin{array}{*{20}l} {{\hat{f}}_{t}} (p) =\underset{{{p}^{\prime}} \in {{\Omega }_{{M}_{m}}}}{\text{median}}\, \left\{f({{p}^{\prime}})\right\} \end{array} $$

where ${{\Omega }_{{M}_{m}}}\phantom {\dot {i}\!}$ is an *M*_*m*_×*M*_*m*_ filter. In this work, we employed median filtering using *M*_*m*_=3.

### Linear renormalization (d)

Variable dynamic range in an image corresponding a particular tag *t*, due for example to different exposure times, may result in biased results. Linear renormalization can be applied to ensure that the dynamic range of each image in the MBI stack is the same.

(4) $$\begin{array}{*{20}l} {{\hat{f}}_{t}}(p) = \frac{{{f}_{t}}(p)-f_{t}^{\min}}{f_{t}^{\max}-f_{t}^{\min}} \end{array} $$

with $f_{t}^{\min }$ and $f_{t}^{\max }$ being the minimum and maximum intensity values of *I*_*t*_.

### Sigmoid renormalization (e)

The strong binding of a protein marker in a particular region may produce high intensity values in that region, rendering the weaker regions almost completely unidentified for analysis purposes due to their relatively weaker intensity. To ensure that weaker signals are enhanced without further enhancing the stronger signals, the hyperbolic tangent function (a scaled form of the sigmoid function commonly used as a neuronal activation function) can be applied to each intensity image [21].

(5) $$\begin{array}{*{20}l} {{\hat{f}}_{t}}(p) = \tanh \left(\frac{1}{2f_{t}^{\text{mean}}}{{f}_{t}}(p)\right) \end{array} $$

with $f_{t}^{\text {mean}}$ being the mean value of *I*_*t*_.

### Principal component analysis (PCA) (f)

After the application of a normalization strategy - made up of clipping (optional), filtering, and renormalization steps - we obtain for each set of MBIs *N* transformed intensity images ${{\left ({{I}_{t}}\in {{\mathbb {D}}^{w\times h}}\right)}_{t=1,\ldots,N}}$. We flatten each image to get column vectors ${{\left (I_{t}^{\prime }\in {{\mathbb {D}}^{(w\cdot h)\times 1}}\right)}_{t=1,\ldots,N}}$. We then define $M\in {{\mathbb {D}}^{(w\cdot h)\times N}}$ as a matrix consisting of N such column vectors. We compute the principal component ${{e}_{\mathbf {i}}}\in {{\mathbb {R}}^{N}}$, regarding each row of *M*as a data point [29].

### Project data (g)

Due to the numeric computation of eigenvectors (i.e., the principal components), the orientation (not to be confused with the direction, accounting for the variance, of course) of principal components *e*_**i**_ may be arbitrary and needs to be aligned to avoid inverted color projections (see below). To ensure that they have a similar direction, we set

(6) $$\begin{array}{*{20}l} {{e}_{\mathbf{i}}}= \left\{ \begin{array}{cc} {{e}_{\mathbf{i}}}, & \text{if}\; {{e}_{\mathbf{i}}}\cdot \vec{1}>0 \\ -{{e}_{\mathbf{i}}}, & \text{otherwise} \\ \end{array} \right. \end{array} $$

After this, *M* is multiplied by an (*w*.*h*)×3 matrix consisting of the first three principal components

(7) $$\begin{array}{*{20}l} \eta =\mathrm{M} \left[{{e}_{1}},{{e}_{2}}, {{e}_{3}} \right],\; \text{where}\; \eta \in {{\mathbb{R}}^{(w.h)\times 3}} \end{array} $$

where *η* can be transformed back to $O\in {{\mathbb {D}}^{w\times h\times 3}}$, resulting in 3 images each having the same size as each of the *N* normalized tag images. Thereafter, *O* is used as an RGB color image after linearly scaling the intensity values in the R, G, and B channels to the domain $\mathbb {D}$. In order to visualize and compare the results generated by the various normalization protocols, we then rescale each of the 3 color channels separately. This way we obtain for each image data set and each normalization strategy a pseudo-color map, representing the variances in the data in relation to tissue morphology and prevalent protein signatures. Pseudo-color visualizations (termed as maps here) have their drawbacks, like for instance the human’s non-homogenous contrast sensitivity along the visual spectrum (i.e. differences between low frequency colors (blue) are not recognized with the same sensitivity as those for higher frequencies (yellow, red)). However, pseudo-color maps can display much more structure in the feature domain than grey value images so they are still a widely used approach to exploratory data analysis in MBI [30].

### Kullback-Leibler (KL) divergence

For quantitative comparison of the normalization protocols we performed Agglomerative Hierarchical Clustering (AGHC) and *k*-means, on average protein expression profile corresponding to each cell in an MBI, to generate cell phenotypes corresponding to histologically normal and cancer samples as described in [19]. To measure the difference between discrete probability distributions of cell phenotyping profile, we employ a symmetric Kullback-Leibler (KL) divergence [31] defined by

(8) $$\begin{array}{*{20}l} KL(P,Q) = \frac{1}{2} \sum\limits_{i\in \mathcal{X}}{(P(}i)-Q(i)) \log \frac{P(i)}{Q(i)} \end{array} $$

where *P* and *Q* are discrete probability distributions on a finite set $\mathcal {X}$ of cell phenotypes in MBI data. According to definition, the KL divergence should be higher when comparing different classes (‘Normal vs Cancer’) whereas it should be low when comparing within the same class (‘Normal vs Normal’ and ‘Cancer vs Cancer’).

## Results

**Fig. 2:**
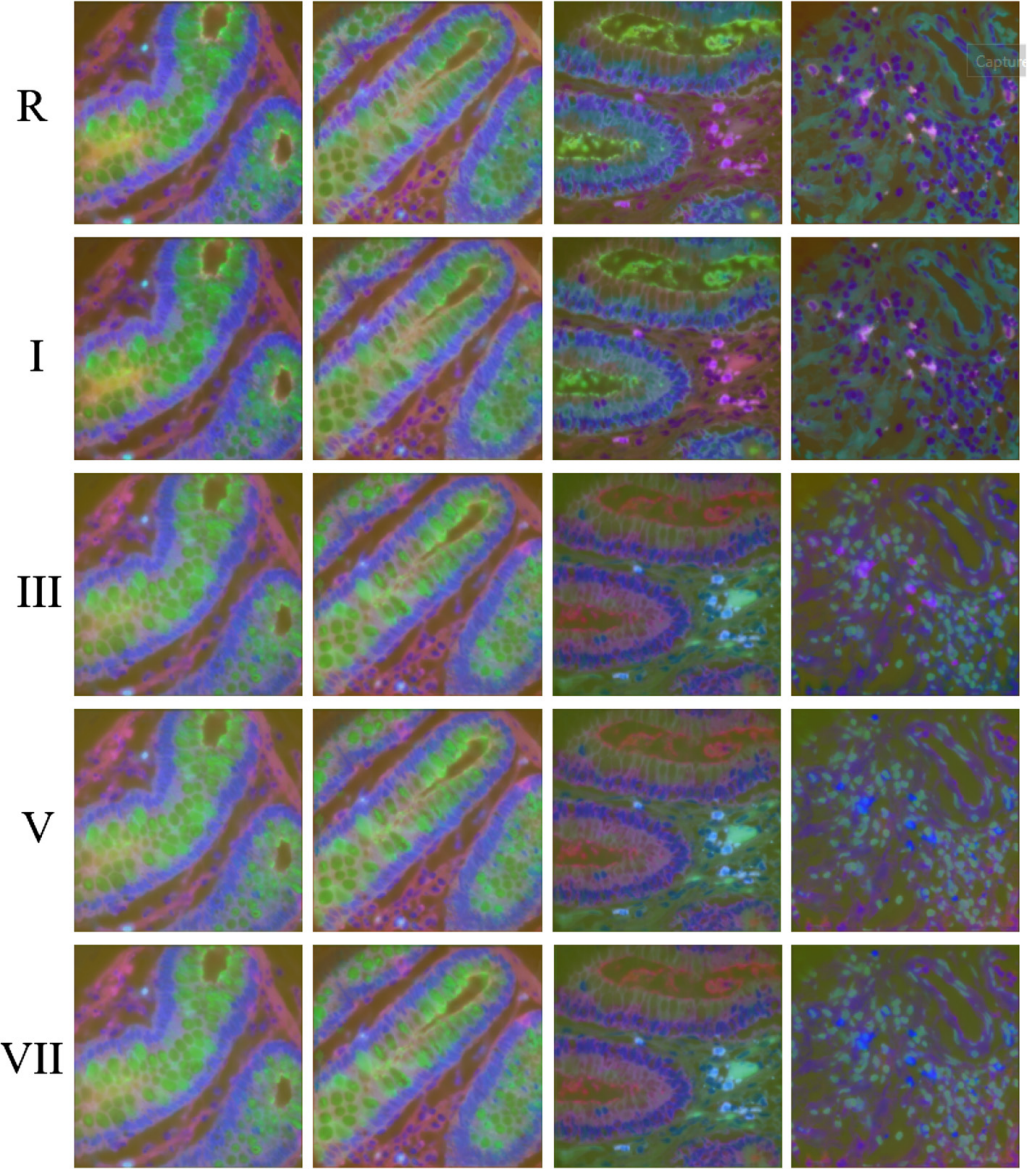
Pseudocolor representation of normalization results. Column 1 to 4 represent four different cases: first two columns are from histologically normal tissue and the last two are from cancerous tissue of the same patient. Row 1 represents pseudo-color image obtained using raw pixel intensity values whereas row 2 to 5 represent psudo-color images obtained after applying different normalization protocols. See the text for details about results shown in I, III, V, VII. The pseudo-color images for the remaining normalization protocols are added in the Additional file 1: Appendix in Figure A-1

To minimize the effect of unknown variations in the data we start our analysis with four MBIs from the same patient, two each of cancerous and adjacent healthy tissue samples. We obtained pseudo-color (section ‘g’) visualizations using all the normalization protocols listed in Table 1 and requested two pathologists and two biologists to rank the results. The results for the top five protocols as ranked by the experts (Table 2) are shown in Fig. 2, whereas the results for the rest of normalization protocols have been added in Additional file 1: Figure A-1. The first two columns represent samples from histologically healthy colon tissue and the last two columns represent samples from cancerous tissue. Before the application of the normalization protocols defined above, the background fluorescence signal was removed by subtracting the auto fluorescence signal image just before applying the respective antibody tag. Two pathologists and two biologists were requested to rank the images based on the criteria A-C (see Materials and methods section above). The experts made following general observations on Fig. 2. Normalization protocols R & I show consistent blue color across the epithelial region in all the three cases where the epithelial cells are well organized around the crypt. Similarly, they shows greenish color inside the crypt and the stromal cells show the purple color in all the four cases. Normalization protocols III, V & VII show consistency in the normal cases but show different colors in the lumen and stromal regions for the cancer cases. Table 2 shows the rank of the normalization protocols as given by the two pathologists (A & B) and the two biologists (C, & D). Three experts ranked normalization protocol I as consistently producing the best results, however one of the experts ranked it as the second best. The reason being that they seemed to be producing very similar results. When results from protocol I and R were carefully examined they seem to produce similar pseudo-color images except that epithelial region in column two show slightly lighter blue for the protocol R compared to epithelial from the protocol I. Experts ‘A’ & ‘C’ preferred the color tone of blue in the protocol I as it was consistent with the color tone in the images in first and third column. Additionally, the protocol I shows higher contrast between signal and the background by suppressing background intensities whereas the protocol R shows slightly higher intensities in the background region. This suggests that the protocol I does not compromise the quality of data while performing normalization. We perform further experiments as explained in the remainder of this section to examine if the protocol I increases the quality of the data and introduces consistency in the results from different runs. Normalization protocols II, IV VI & VIII, (Figure A-1 in Additional file 1: Appendix) do not exhibit color consistency in all four cases and were ranked low by experts. For example, all of these protocols show a variation in color in the epithelial cells around the crypt. Even in the healthy cases, the colors are not stable and show a lot of variation. The same is the true for the lumen areas, goblet cells and the stromal cells.

**Table 2:** Rank given to normalization protocols R, I to VIII by two pathologists (A & B) and two biologists (C & D). The rows represent ranks given by each of the four experts whereas the columns represent rank of an MBI

Expert	1	2	3	4	5	6	7	8	9
A	I	R	VII	V	III	IV	II	VIII	VI
B	I	R	V	II	IV	VII	VIII	VI	III
C	I	R	VII	V	III	II	IV	VIII	VI
D	R	I	VII	III	V	II	IV	VIII	VI

Another interesting feature of protocol R & I is the particular foreground / background contrast of one specific cell, which can be seen in the upper-left quadrant of images in the first column (Fig. 2). It appears as a small blue / cyan dot. An in-depth analysis has shown that this cell expresses an unusual combination of almost all proteins tagged in this experiment. Such rare occurrences could yield potential cues to rare events in the specimen, such as cancer stem cells. The contrast of this particular cell is high for protocols R, I, III, and V and ideal for protocol I and R.

**Fig. 3:**
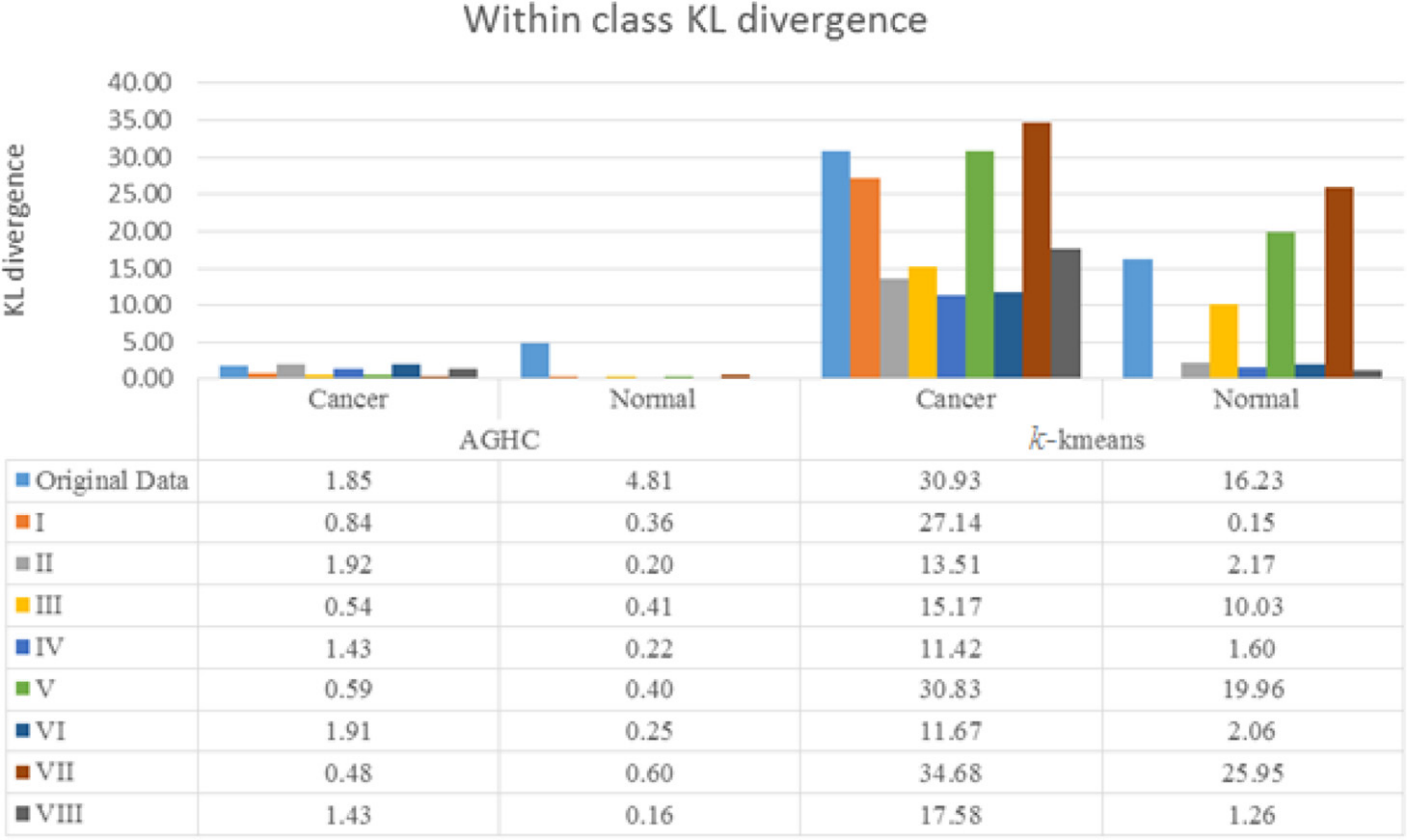
Within class KL divergence after applying different normalization protocols. Normalization protocol I shows lower KL divergence in all cases except for *k*-means clustering on cancer data

**Fig. 4:**
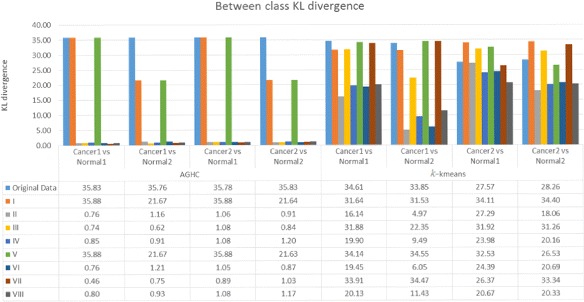
Between class KL divergence after applying different normalization protocols. Only R, I & VII show higher KL divergence while performing AGHC & *k*-means

To compute KL divergence, distribution of cell phenotypes obtained using the method proposed in [19] was compared in normal and cancer samples. Figure 3 shows results for within class KL divergence, whereas Fig. 4 shows between class KL divergence results for normal and cancer samples, where Normal1 and Normal2 correspond to columns 1 and 2, whereas Cancer1 and Cancer2 refer to columns 3 and 4 respectively in Fig. 2. We expect lower within class KL-divergence as the same class should exhibit similar phenotypes whereas higher between class KL-divergence as different classes should exhibit different phenotypes. Figure 4 shows that only R, I and V produced higher KL divergence whereas the rest of the normalization protocols failed to show separation between the phenotypes while performing AGHC. Protocol R shows higher KL divergence in both Normal2 vs Cancer1 and Normal2 vs Cancer2 cases compared to protocol I and V which is desired, but it also shows higher within class KL divergence for Normal when performing AGHC. When Normal2 is carefully observed in Fig. 2, the stromal cells show a variation in colour within the same image for protocol R as can be seen in the stromal cell at the bottom of the image (Row 1, Column 2). Protocol I and V do not suffer from this discrepancy. Similarly, within class KL-divergence for *k*-means show higher values for protocol R & V. Compared to protocol R normalization protocol I shows lower within class KL divergence in all *k*-means cases. However, normalization protocol I shows higher KL divergence compared to II,III, IV, VI, VIII normalization protocols while performing phenotyping using *k*-means clustering on cancer data. This is likely due to the difference in histologic grade of the cancer tissues. The normal tissues on the other hand show very low KL divergence for protocol I. Protocol I shows higher KL divergence for all the cases except for *k*-means Cancer1 vs Normal1 and Cancer1 vs Normal2, but these values are very close to the ones obtained using protocol R. Protocol R on the other hand shows lower values for KL divergence for the *k*-means Cancer2 vs Normal1 and Cancer2 vs Normal2. Results obtained using protocol VII and IV can be studied in a meaningful way when the results from these protocols are combined in Figs. 3 and 4. Protocol VII shows higher between class KL divergence but it also shows higher KL divergence for within class KL divergence in Fig. 3. Similarly, protocol IV shows lower values in Fig. 3 but it also lowers the between class KL divergence.

We performed the same experiment with three MBIs collected from another patient, which contains one MBI from cancer sample and two MBIs from adjacent histologically normal samples. We have added the results in the Additional file 1: Appendix for within class KL-divergence in Additional file 1: Figure A-2 and for between class KL divergence in Additional file 1: Figure A-3. The KL-divergence result show similar kind of pattern for protocols R, I & V as in Figs. 3 and 4 respectively. The protocol R shows higher within class KL-divergence for normal samples with *k*-means clustering. However, for this patient, protocol VII behaved differently and shows higher between class KL-divergence when performing clustering using AGHC, but between class KL-divergence is lower for protocol VII when performing clustering using *k*-means, showing inconsistency in the results.

In addition to above experiments, we combined data from four cancer MBIs and six histologically normal MBIs and calculated KL-divergence on the cell phenotypes obtained using AGHC and *k*-means clustering. For computing between class KL-divergence (i.e., Cancer vs Normal (CN)), we generate a ‘normal’ mosaic using six histologically normal MBIs and a ‘cancer’ MBI mosaic using four cancer MBIs and perform clustering to obtain cell phenotypes. For between class KL-divergence, i.e., normal vs normal (NN) and cancer vs cancer (CC), we generate the mosaic by dividing each MBI into two halves and use one half to contribute to artificially generated one mosaic and the other half artificially generated second mosaic. In this way we can make sure that we are not missing any cell phenotypes which might be present in one patient and not in another. At the same time, by using half of the image we create separation between the data in a way that the data is not taken from the same region. The results for KL-divergence are shown in Table 3, which shows that protocol I shows lower within class KL-divergence and higher between class KL-divergence. In the case of *k*-means protocol V shows higher between class KL-divergence but at the same time it has higher within class KL-divergence. Also, for in the AGHC case, protocol V produces lower between class KL-divergence. Therefore, there is inconsistency in the results as evident from results in Fig. 3 and Additional file 1: Figure A-2 which shows higher within class KL-divergence for protocol V. Similarly, protocol R shows higher within class KL-divergence for *k*-means Normal case both in Fig. 3 and Additional file 1: Figure A-2. Normalization protocol I as ranked by majority of the experts increases the separation between clusters when comparing different classes but decreases this separation within the same class. The consistency of protocol I makes it the best choice for normalization among the comparable schemes.

**Table 3:** KL-divergence result for the mosaic image created using multiple MBIs

	*k*-means			AGHC		
	CC	NN	CN	CC	NN	CN
R	0.43	0.51	5.97	0.13	0.32	0.56
**I**	**0.26**	**0.22**	**8.37**	**0.25**	**0.27**	**0.58**
II	0.16	0.48	0.42	0.13	0.15	0.14
III	0.52	0.19	2.08	0.09	0.12	0.22
IV	0.16	0.42	0.46	0.10	0.14	0.21
V	0.46	0.33	10.08	0.25	0.27	0.21
VI	0.26	0.35	0.36	0.13	0.13	0.13
VII	0.52	0.16	1.22	0.07	0.14	0.13
VIII	0.22	0.43	0.66	0.08	0.16	0.13

CC represents cancer vs cancer, NN represents normal vs normal and CN represents Cancer vs Normal KL-divergence. Protocol I (bold) produces high inter-class divergence while simultaneously preserving low intra-class divergence

## Conclusions

Standardization of normalization procedures for data acquired from multiplexed bioimaging (MBI) technologies is as important as the standardization of protocols for the preparation of the tissue. This is mainly because of the presence of inherent limitations of the imaging apparatus, which can be due to variations in quality, quantity, or concentration of the antibody tag, exposure times, and quality of the camera and the microscope being used. Although efforts are being made to optimize the procedure for data acquisition and preparation of slides under the microscope [23], normalization of the data, i.e. the alignment of signals will always be necessary to overcome the variation across various runs for different types of tissue. Normalization protocols have been attempted in the past for other multiplexed technologies such as MALDI, mainly based on heuristics [24]. We presented a normalization pipeline for the normalization of MBI data and compared the performance of its eight variants for data sets collected from ten different tissue samples, six histologically healthy and four cancerous samples. Three of the four experts, two pathologists and two biologists, agreed on the normalization protocol I (made up of bilateral filtering followed by linear scaling) to be performing the best, whereas one expert ranked protocol I to be second best.

Protocol I also ranks best in terms of consistency in KL-divergence results, and is a combination of no clipping, bilateral filtering and linear normalization. Using protocol I, different constituents in the tissue, for example epithelial tissue, lumen and stromal cells produced consistent visualization across all the images from different types of tissue. In addition, normal and cancer tissues produced desired results after calculating KL divergence on cell phenotypes. The results suggest that if images do not contain over saturated intensities, clipping may destroy the quality of the data in those images. Bilateral filtering denoises the images but does not merge different compartments of the tissue as does the conventional median filtering. Linear scaling linearly stretches the intensities from 0 to 1 (maximum intensity), for all the protein expressions, therefore dynamic range of expression of protein intensities is preserved across different runs, while the results suggest that non-linear sigmoid scaling degrades the quality of data. It seems that linear scaling has major impact on the normalization protocol as R, I, III, V & VII rank best by expert markings but if the results are studied in detail it is the combination of bilateral + linear normalization which makes it the best normalization protocol. If bilateral filtering is replaced with median as in protocol V (No clipping + median + linear), protocol V shows different visualization for stromal cells in Fig. 2, in addition the results show higher within class KL-divergence in Fig. 3 and Additional file 1: Figure A-2. This suggests that it is the combination of No clipping + bilateral filtering + linear scaling which produces the best results.

## Additional file

Additional file 1
**Appendix.** Figure **A-1**: Column 1 to 4 represent four different cases: first two columns are from histologically normal tissue and the last two are from cancerous tissue of the same patient. Rows 1 to 4 represent pseudo-color images obtained after applying low rank normalization protocols as marked by the experts. Figure **A-2**: Within class KL-divergence for Patient 2. Within class KL-divergence for second patient after performing phenotyping using different normalization protocols. Figure **A-3**: Between class KL-divergence for Patient 2. Between class KL-divergence for second patient after performing phenotyping using different normalization protocols. (PDF 341 kb)

## Abbreviations

TIS: toponome imaging system
MBI: multiplexed bioimaging data
KL: Kullback-Leibler
CMP: combinatorial molecular patterns
