# Supplementary material for: Robust normalization protocols for multiplexed fluorescence bioimage analysis
Source: BioData Min. 2016 Mar 5;9:11. doi: 10.1186/s13040-016-0088-2 (PMC4779207; doi:10.1186/s13040-016-0088-2)
Supplement: Additional file 1 — Appendix. Figure A-1: Column 1 to 4 represent four different cases: first two columns are from histologically normal tissue and the last two are from cancerous tissue of the same patient. Rows 1 to 4 represent pseudo-color images obtained after applying low rank normalization protocols as marked by the experts. Figure A-2: Within class KL-divergence for Patient 2. Within class KL-divergence for second patient after performing phenotyping using different normalization protocols. Figure A-3: Between class KL-divergence for Patient 2. Between class KL-divergence for second patient after performing phenotyping using different normalization protocols. (PDF 341 kb) [file 13040_2016_88_MOESM1_ESM.pdf]

Appendix

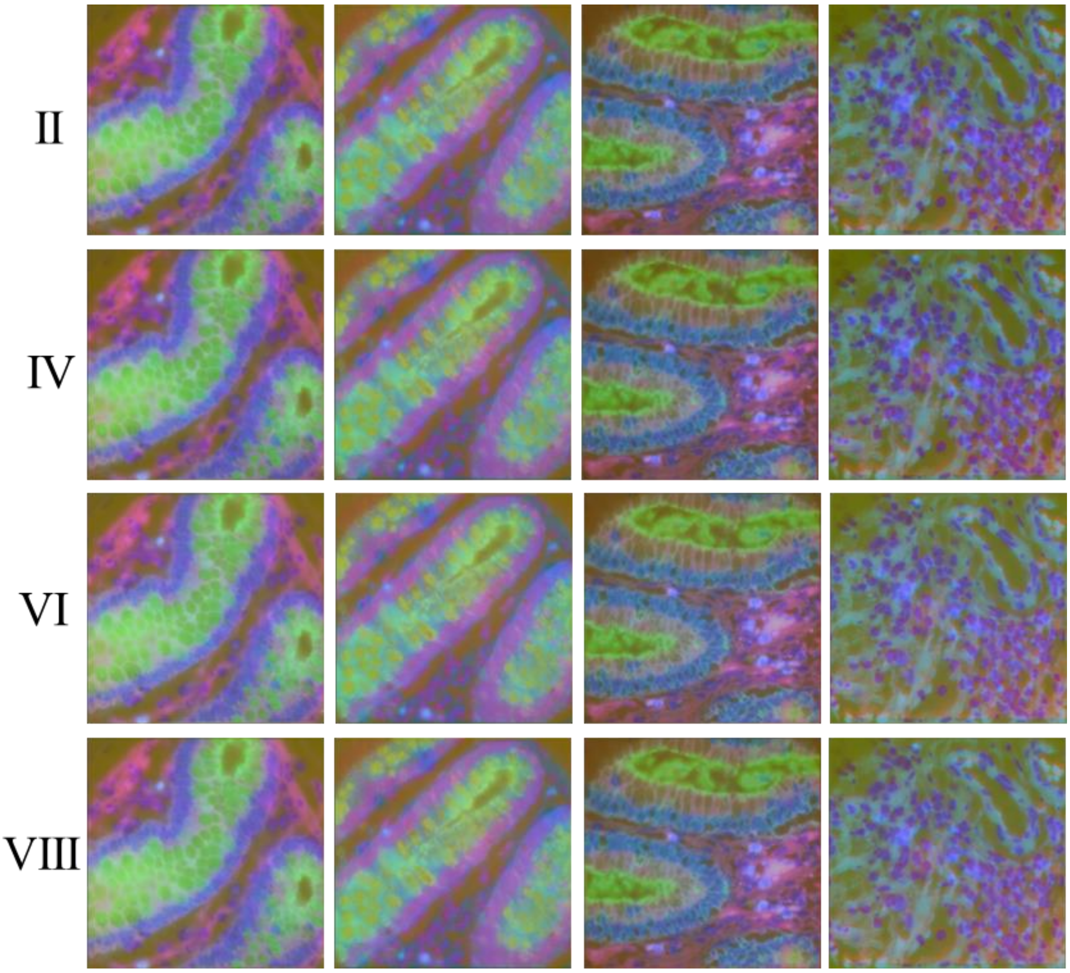

Figure A- 1: Column 1 to 4 represent four different cases: first two columns are from histologically normal tissue and the last two are from cancerous tissue of the same patient. Rows 1 to 4 represent pseudo-color images obtained after applying low rank normalization protocols as marked by the experts.

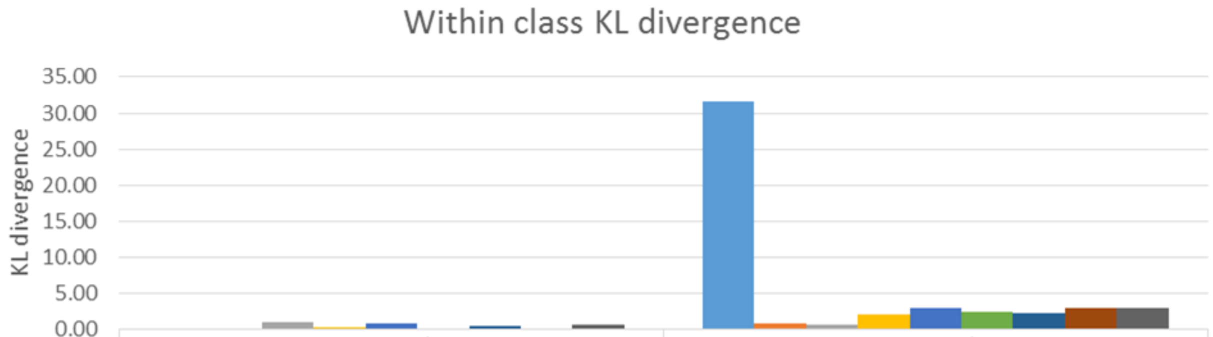

	Normal AGHC	Normal *k*-means
R	0.23	31.58
I	0.19	0.95
II	1.06	0.72
III	0.40	2.09
IV	0.80	2.93
V	0.19	2.51
VI	0.57	2.35
VII	0.21	2.93
VIII	0.78	2.99

Figure A- 2: Within class KL-divergence for Patient 2. Within class KL-divergence for second patient after performing phenotyping using different normalization protocols.

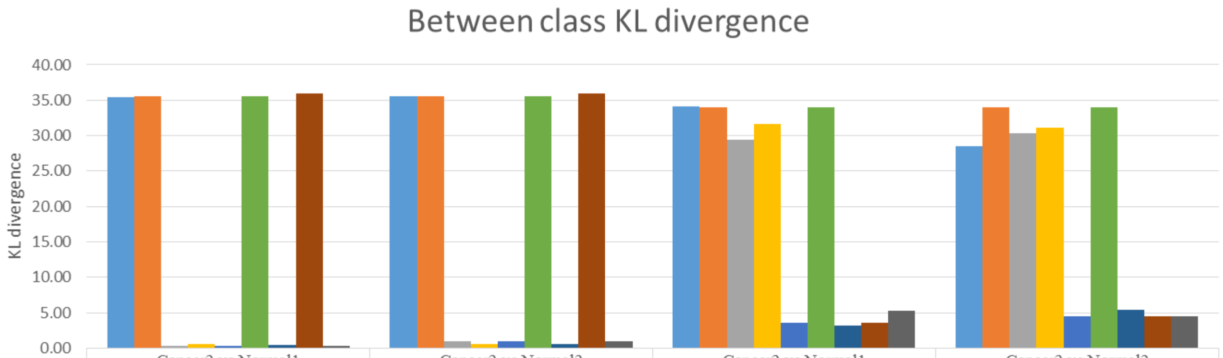

	AGHC		*k*-means	
	Cancer2 vs Normal1	Cancer2 vs Normal2	Cancer2 vs Normal1	Cancer2 vs Normal2
R	35.47	35.58	34.18	28.44
I	35.51	35.59	34.00	33.94
II	0.37	0.97	29.45	30.35
III	0.54	0.55	31.66	31.06
IV	0.31	0.90	3.53	4.50
V	35.51	35.59	34.00	33.97
VI	0.39	0.51	3.22	5.36
VII	35.90	35.89	3.53	4.50
VIII	0.34	0.91	5.28	4.43

Figure A- 3: Between class KL-divergence for Patient 2. Between class KL-divergence for second patient after performing phenotyping using different normalization protocols.
